# Graphene Oxide Electroreduced onto Boron-Doped Diamond and Electrodecorated with Silver (Ag/GO/BDD) Electrode for Tetracycline Detection in Aqueous Solution

**DOI:** 10.3390/nano11061566

**Published:** 2021-06-14

**Authors:** Sorina Negrea, Lidia Ani Diaconu, Valeria Nicorescu, Sorina Motoc (m. Ilies), Corina Orha, Florica Manea

**Affiliations:** 1 National Institute of Research and Development for Industrial Ecology (INCD ECOIND), 300431 Timisoara Branch, Romania; negrea.sorina@yahoo.com (S.N.); lidia.diaconu29@yahoo.com (L.A.D.); nvaleriarus@yahoo.com (V.N.); 2 Department of Environmental Engineering and Management, “Gheorghe Asachi” Technical University of Iasi, 700050 Iasi, Romania; 3 “Coriolan Dragulescu” Institute of Chemistry, Romanian Academy, Mihai Viteazul 24, 300223 Timisoara, Romania; sorinailies@acad-icht.tm.edu.ro; 4 National Condensed Matter Department, Institute for Research and Development in Electrochemistry and Condensed Matter, Timisoara, 1 P. Andronescu Street, 300254 Timisoara, Romania; orha.corina@gmail.com; 5 Faculty of Industrial Chemistry and Environmental Engineering, Politehnica University Timisoara, P-ta Victoriei No. 2, 300006 Timisoara, Romania

**Keywords:** silver electrocatalyst, tetracycline, electrochemical detection, silver-graphene oxide-boron-doped diamond

## Abstract

A new electrochemical sensor designed by modifying the commercial boron-doped diamond (BDD) electrode with graphene oxide (GO) reduced electrochemically and further electrodecorated with silver (Ag), named the Ag/GO/BDD electrode, was selected among a series of the BDD, GOelectroreduced onto BDD (GO/BDD) and silver electrodeposited onto BDD (Ag/BDD) electrodes for the detection of tetracycline (TC) in aqueous solution. The best results regarding the sensitivity of 46.6 µA·µM^−1^·cm^−2^ and the lowest limit of detection of 5 nM was achieved using square-wave voltammetry (SWV) operated at the step potential of 5 mV, modulation amplitude of 200 mV and the frequency of 10 Hz in alkaline medium. The application of the alkaline supporting electrolyte-based procedure is limited for water monitoring due to the presence of chloride that interferes with TC detection; however, it can be applied for quantitative determination of pharmaceutical formulations. 0.1 M Na_2_SO_4_ supporting electrolyte eliminated chloride interference and can be used for the application of Ag/GO/BDD in practical detection of TC in water.

## 1. Introduction

Environmental pollution created by antibiotics is considered a serious problem, although no toxic reports have been known so far, but they are present in the environment and categorized as “emerging pollutants”. Among antibiotics, tetracycline (TC) is one of the main groups of antibiotics used for veterinary purposes, for human therapy and for agricultural purposes, which represent the main sources of environmental pollution. Recently, it has been reported that the appearance of tetracyclic antibiotics in the environment inhibits the growth of terrestrial and aquatic species [1,2]. TC is a broad-spectrum polychid antibiotic that has been widely used for more than 50 years in the treatment of bacterial infections in both humans and animals [3,4]. Being an effective antibiotic, the abuse of TC in animal husbandry and aquaculture results in a high level of pollution in terms of soil and water, thus, it is considered an important organic contaminant present in water. Its degradation is difficult and it remains active in water, thus leading to negative effects on ecosystems and human health. Some data have been reported concerning the occurrence of TCs in various aquatic environmental matrixes, such as: seawater (1.55–2500 ng·L^−1^), lakes (2.7–70 ng·L^−1^), rivers (1.1–54.000 ng·L^−1^), groundwaters (9.51–29.700 ng·L^−1^), drinking water (11.2–632 ng·L^−1^) and wastewater (0.0019–129.3 ng·L^−1^) [5].

In this context, the development of effective platforms for both detection, monitoring and removal of TC in water is highly desirable, becoming a challenge for many researchers [3]. Currently, the detection and quantification of tetracycline is based on traditional analytical methods, such as: high-performance liquid chromatography (HPLC), liquid chromatography coupled with tandem mass spectrometry (LC-MS), ultraviolet (UV) detection and capillary electrophoresis (CE) [4]. Even if these techniques have high sensitivity and accuracy, they are time consuming and require expensive and complicated equipment, thus limiting their application in routine analysis. Also, these methods require the use of potentially harmful solvents, the need for training, and certified operators. Inevitably, these methods are limited in terms of on-site, instantaneous and in situ analyses [6]. Although chromatographic procedures usually offer lower detection limits, electrochemical methods can be an interesting alternative due to their simplicity, short operating time, low cost and small amount of waste generation [1,7].

The ease of electrochemical methods in operation, rapid analytical response, absence of pretreatment sample, technically miniaturized and portable devices, make them accessible for on-site analysis [8,9,10].

According to recent studies reported in the literature, the use of electrochemical methods as well as electrochemical sensors have allowed the electrochemical detection of tetracycline at values of 0.360 μM using a carbon paste electrode modified with a combination of multiwalled carbon nanotubes functionalized with carboxyl groups (MWCNT-COOH) [11], 0.160 μM TC using a benzene sourced graphene-gold nanoparticle sensor for the detection of tetracycline (GC Gr-AuNP) modified electrode [12], and 15.2 μM TC using multisegment nanoparticles [13].

The performance of these electrochemical methods is determined by the electrode material and electrochemical techniques. The use of standard sensors during pollutant analysis is prone to some disadvantages, including electrode passivation, excessively high potential of analyte reactions [14,15,16]. However, there are mitigation strategies against these limitations, thus improving the sensitivity and accuracy of the sensors. Electrode modification is such an approach, using various materials: carbon-based materials, NP metals (oxides), polymers, etc. The modification of the sensors serves also to decrease the excessive potential of electrochemical reactions, the preconcentration capacity for some analytes, generating electrode modification interfaces that ensure the formation of bridges and pathways to allow electron transfer to ultimately improve signal amplification [17,18,19]. Carbon-based materials have remarkable characteristics, such as: high surface area, high electrical conductivity, electron mobility at room temperature, flexibility and mechanical strength, making them very attractive in the field of sensor design. Of these carbonaceous materials, due to its electrical conductivity and very high surface area, graphene is ideal for electrochemical applications [20,21,22,23]. The boron diamond electrode (BDD) is recognized for its outstanding properties in very good results in electrochemical detection and electrochemical applications, such as: reproducibility, low background current, wide potential window, stability. By modifying the electrode surface with nanostructured material, for example graphene, the electrocatalytic effect and the electroactive surface are enhanced. Also, by silver particles deposited on the BDD electrode, the electrocatalytic activity is reflected by the higher rate of transfer electrons and by the improvement of sensitivity regarding the electrochemical detection of the target analytes [24,25,26].

In the present paper, the synergistic effect of the combination of graphene and silver particles on the BDD electrode as support material will be tested in comparison with graphene-modified BDD, silver modified BDD and unmodified BDD electrode for the detection of tetracycline, an emerging pollutant and its performance with respect to sensitivity and the lowest limit of detection is evaluated and discussed.

## 2. Materials and Methods

A scanning electron microscope (SEM) using the Inspect S PANalytical model (FEI Co., Eindhoven, The Netherlands) coupled with an energy-dispersive X-Ray analysis detector (EDX) was used to characterize the morphology of the modified electrode surface, using catalyst powder supported on carbon tape.

The electrochemical experiments were performed using an Autolabpotentiostat/galvanostat PGSTAT 302 (Eco Chemie, Utrecht, The Netherlands), with a standard three electrode cell, using three types of BDD-based electrodes. A boron-doped diamond electrode modified with graphene oxide (GO) reduced electrochemically, and further decorated with silver, named Ag/GO/BDD, and BDD decorated with silver (Ag/BDD) were used in comparison with graphene oxide electrochemically reduced onto BDD (GO/BDD) and BDD electrodes as working electrodes. A platinum counter-electrode and a saturated calomel electrode (SCE) was used as the reference electrode. The BDD commercial electrode provided by Metrohm (Herisau, Switzerland), characterized by a disc surface with the 3 mm diameter, mechanically cleaned using 0.2 μm alumina powder (Al2O3), and then washed with distilled water. The electrochemical modification of the BDD electrode with graphene oxide occurred by an electrochemical deposition process at the potential of −1.50 V for 120 s using a chronoamperometry (CA) technique, from a suspension of 4 mg/mL GO dispersed in water. After the graphene electrodeposition, the silver particles (AgPs) decorated the electrode surface by electrodeposition at −1.30 V/SCE for 5 s from the 4 mM AgNO3 solution.

The target analyte solution, tetracycline, was provided by Antibiotics (Romania) and the stock solution of 1 mM was prepared by using 0.1 M NaOH solution (Merck, Germany). Before each experiment, the working electrode was electrochemical stabilized through 10 continuous repetitive cyclic voltammograms within the potential ranging between −0.50 and +1.00 V/SCE in 0.1 M sodium hydroxide (NaOH) solution prepared using analytical-grade reagent from Merck which was used as a supporting electrolyte; a 0.1 M Na_2_SO_4_ supporting electrolyte prepared from analytical grade reagent from Merck (Darmstadt, Germany) was also used for the study. Cyclic voltammetry (CV), differential-pulsed voltammetry (DPV), square-wave voltammetry (SWV), chronoamperometry (CA) and multiple-pulsed amperometry (MPA) were used to determine the electroanalytical performance of the BDD-based electrodes for TC detection. The lowest limit of detection (LOD) was calculated using the equation: LOD = 3SD/m and LOQ = 10SD/m, where SD is the standard deviation of threeblanks and m is the slope of the analytical plots [26].

## 3. Results

### 3.1. Morpho-Structural Characterization

**Figure 1:**
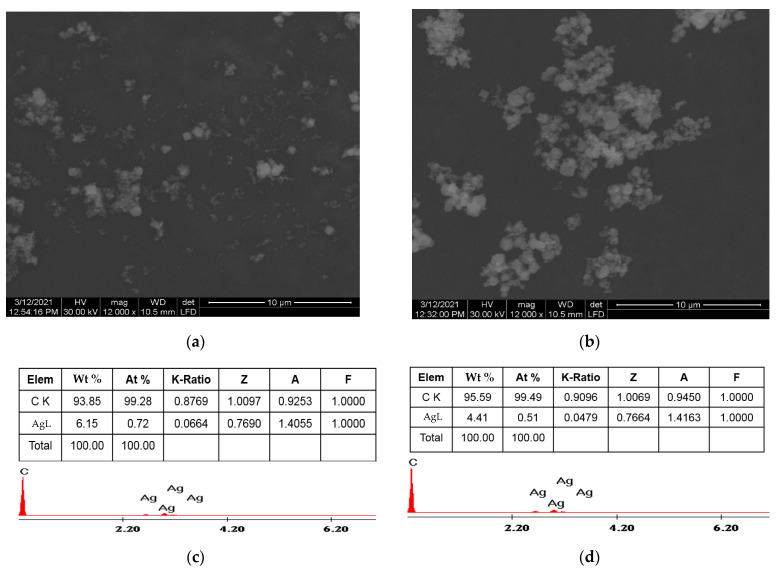
Scanning electron microscopy (SEM) images of: silver/boron-doped diamond (Ag/BDD)(**a**);silver/graphene oxide/boron-doped diamond(Ag/GO/BDD)(**b**); energy-dispersive X-ray spectroscopy (EDX) images of Ag/BDD (**c**); Ag/GO/BDD (**d**).

Figure 1a,b shows the SEM images of Ag/BDD, and Ag/GO/BDD electrode surfaces and EDX images are presented in Figure 1c,d. The presence of irregular particles, which exhibit an obvious tendency to agglomerate,and a non-homogeneous dispersion of silver particles onto carbon surfaces, can be noted.

### 3.2. Cyclic Voltammetric Measurements

The effect of the Ag electrodeposition including graphene integration onto the BDD electrode surface were studied through the electroactive surface area modification and TC detection response. First, before testing the TC response, the electrochemical surface areas for all studied electrodes were determined considering its importance in any electrochemical response.

CVs recorded at Ag/BDD and Ag/GO/BDD in comparison with GO/BDD and BDD electrodes, in the presence of 4 mM K_3_[Fe(CN)_6_] in 1 M KNO_3_ supporting electrolyte at different scan rates, allowed us to determine the apparent diffusion coefficient in according to the Randles–Sevcik Equation (1):

(1) $$I_{p}=2.69\times 10^{5}AD^{1/2}n^{3/2}v^{1/2}C$$

where *A* represents the area of the electrode (cm^2^), *n* the number of electrons participating in the reaction (equal to 1), *D* the diffusion coefficient of the molecule in solution, *C* the concentration of the probe molecule in the solution which is 4 mM, and *v* is the scan rate (V·s^−1^). Considering the theoretical diffusion coefficient value of 6.70·10^−6^·cm^−2^·s^−1^reported in the literature data [27], the values of the electroactive electrode areas were determined and gathered in Table 1.

It was found that silver decoration and graphene integration increased slightly the electroactive surface area, which are lower in comparison with the geometrical one (0.09 cm^2^).

Selection of the Working Electrode for Tetracycline (TC)Detection

**Figure 2:**
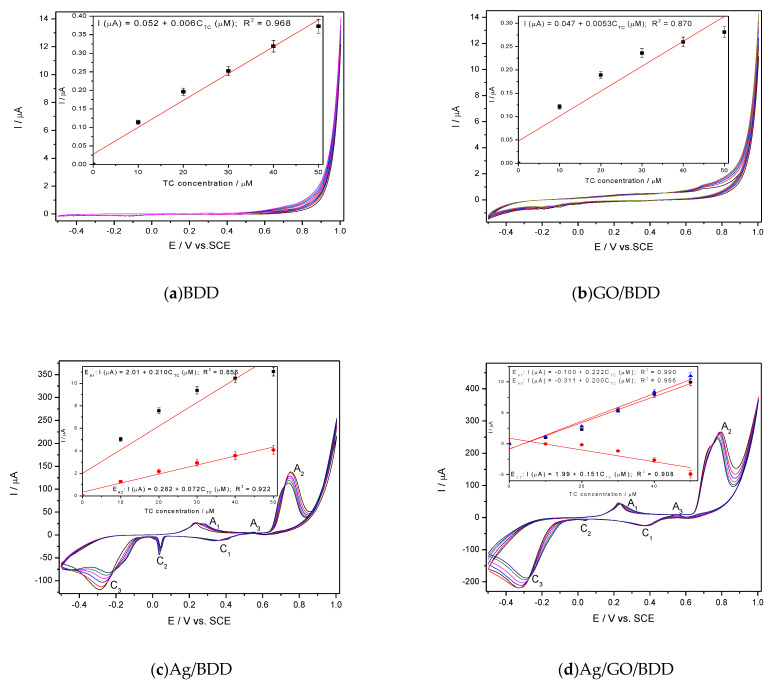
Cyclic voltammetry (CV) recorded at the scan rate of 0.05 V·s^−1^ in 0.1M NaOH supporting electrolyte and tetracycline (TC) concentrations ranged from 10 to 50 µM on the electrode: BDD (**a**); GO/BDD (**b**); Ag/BDD (**c**) and Ag/GO/BDD (**d**); Insets: Calibrations plots of peak current vs. TC concentrations at the potential value specific to each electrode: E = +0.700V/SCE (saturated calomel electrode) (**a**); E = +0.700 V/SCE (**b**); E_1_ = +0.285 V/SCE, E_2_ = +0.550 V/SCE, E_3_ = +0.670 V/SCE (**c**); E_1_ = +0.039 V/SCE (cathodic), E_2_ = +0.296 V/SCE, E_3_ = +0.550 V/SCE, E_4_ = +0.670 V/SCE (**d**).

Considering the potential for silver oxides formation onto the electrode surface, 0.1 M NaOH was used as supporting electrolyte for all studied electrode compositions. The comparative responses for TC detection of the modified BDD electrodes were determined by CV within the TC concentrations ranging from 10 to 50 µM. A series of CV recorded at each electrode are presented in Figure 2a,b considering the calibration plots of the peak current versus TC concentrations, included as insets.

The electrochemical deposition of graphene onto the BDD electrode surface influenced the background current through capacitive component but not the anodic peak characteristic to the TC oxidation onto the carbon surface in comparison with a simple BDD electrode (Figure 1a,b). Similar responses for TC detection were found for GO/BDD and BDD electrodes (see insets of Figure 1a,b and Table 1). Silver electrodeposition onto the BDD electrode changed significantly the CV shape through peaks corresponding to silver redox systems involved in the oxidation/reduction of TC and its oxidation product as well. Three anodic peaks prior to oxygen evolution (A_1_, A_2_, A_3_) and three cathodic peaks (C_1_, C_2_, C_3_) can be seen on the CV series recorded at both Ag/BDD and Ag/GO/BDD in 0.1M NaOH (Figure 1c,d). In according with the literature [28], the anodic peak A1 can be ascribed to the electroformation of Ag_2_O and the anodic peak A2 corresponds to the Ag_2_O electrooxidation to AgO according to the reaction:

2Ag + 2OH^−^ → Ag2O + H_2_O + 2e^−^ (2)

Ag_2_O + 2OH^−^ → 2AgO + H_2_O + 2e^−^ (3)

In addition, it is possible that the AgOis formed based on the reaction:

Ag + 2OH^−^ → AgO + H_2_O + 2e^−^ (4)

Very interesting behaviour was found during the reverse scanning through the existence of an anodic peak (A_3_) that can be attributed to a continuous nucleation of Ag_2_O. The cathodic peak C_1_ is ascribed to the electroreduction of AgO to Ag_2_O and the others two more to the the complex nature of the Ag_2_O electroreduction [25]. It is very interesting that by adding TC, only A_1_ and A_3_ anodic peaks increased linearly with TC increasing, which shows that Ag_2_O is involved in TC oxidation. Also, cathodic peak C_2_ increased cathodically with TC concentration for both Ag-based electrodes but only the presence of graphene assured a linear increasewith TC concentrations for Ag/GO/BDD electrode, while for Ag/BDD electrode a non-linear increasewas noticed. This should be explained by the surface-controlled processes that affected the Ag_2_O involved electroreduction process. Based on the aforementioned CV results, the sensitivity to TC detection for each electrode is presented in Table 2, and it can be noticed that the best characteristics related to the sensitivity and the detection potentials were obtained for Ag/GO/BDD electrode, which was selected for further studies.

### 3.3. Silver/Graphene Oxide/Boron-Doped Diamond(Ag/GO/BDD)Characterization and Testing in TC Detection

#### 3.3.1. Influence of the Scan Rate

**Figure 3:**
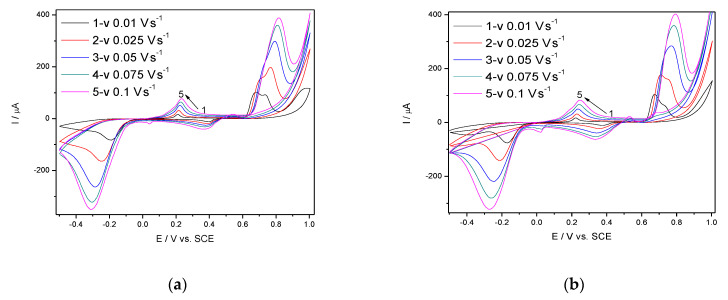
CVs recorded at Ag/GO/BDD electrode with various scan rates ranged from 0.010 to 0.100 V·s^−1^ in: 0.1 M NaOH supporting electrolyte (**a**) and 40 µM TC (**b**).

Considering the results above, Ag/GO/BDD was characterized in 0.1 M NaOH supporting electrolyte and 40 µM TC by the influence of the scan rate. CVs recorded at Ag/GO/BDD at the various scan rates ranged from 0.010 to 0.100 V·s^−1^and are presented in Figure 3a,b.

**Figure 4:**
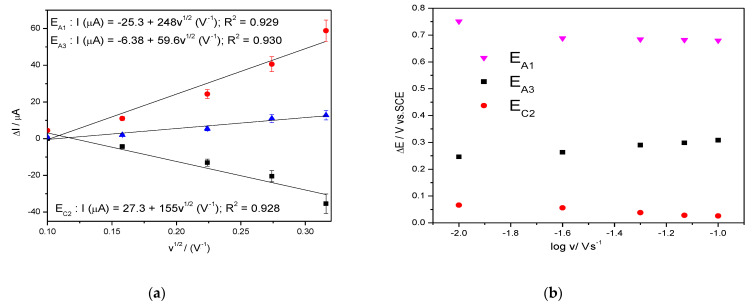
Dependence of the anodic peak currents vs. square root of the scan rate (**a**); evolution of the detection potential values vs. the logarithm of the scan rate (**b**).

The shapes of the CVs are similar in the absence/presence of TC but the anodic current is higher in the TC presence at the detection potentialsestablishedabove, where Ag_2_O is involved. The dependence of the anodic current from which the background current is subtracted vs. the square root of the scan rate is shown in Figure 4a and the dependence of the detection potential values with the logarithm of the scan rate is presented in Figure 4b.

#### 3.3.2. Testing Ag/GO/BDD Electrode for TC Detection in Tap Water Using Cyclic Voltammetry(CV)

**Figure 5:**
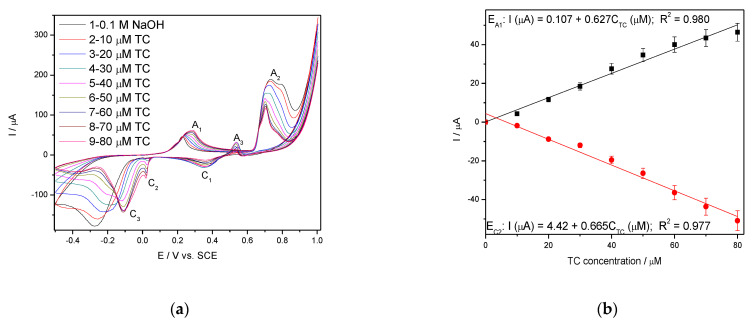
(**a**) CVs recorded at the scan rate of 0.05 V·s^−1^ in 0.1M NaOH supporting electrolyte and TC concentrations ranged from 10 to 80 µM on the electrode Ag/GO/BDD. (**b**) Calibrations plots of peak current vs. TC concentrations at the potential value: E_A1_ = +0.287 V/SCE (anodic), E_C2_ = +0.020 V/SCE (cathodic).

**Figure 6:**
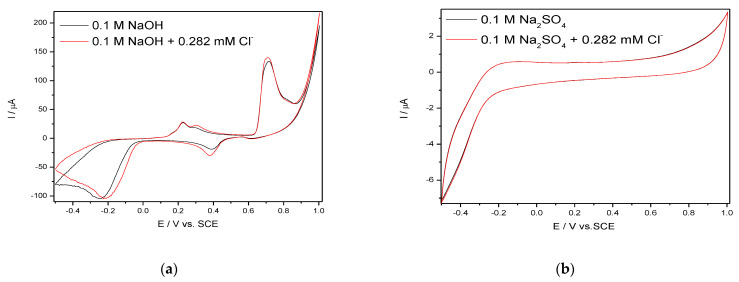
CVs recorded at the scan rate of 0.050 V·s^−1^ in the presence of 0.282 mM Cl^−^ in the supporting electrolyte: 0.1M NaOH (**a**) and 0.1M Na_2_SO_4_ (**b**).

Considering the practical application of Ag/GO/BDD electrode in TC detection in real tap water, its electrochemical behaviourat the increased concentrations of TC in water was studied and the series of the voltammograms recorded in the presence of tap water spiked with the increasing TC concentrations similar with aforementioned concentration range are presented in Figure 5a. The electrochemical behaviour of Ag/GO/BDD electrode is changed in tap water. The electrochemical signals are recorded for anodic peak A_1_ and the cathodic peak C_2_ and the signals are about three times better than in simple 0.1 M NaOH (Figure 5b). Also, no electrochemical signal for TC detection was found at A3 peak due to the anodic peak not increasinglinearly at increasing TC concentration. This behaviour is due to the presence of Cl^−^ that react with Ag(I) oxide and interfere the TC detection. This is proved by the recording CV comparatively in 0.1 M NaOH and in the presence of 0.282 mM Cl^−^, chosen based on the tap water composition in relation with the Cl^−^ content (Figure 6a).

**Figure 7:**
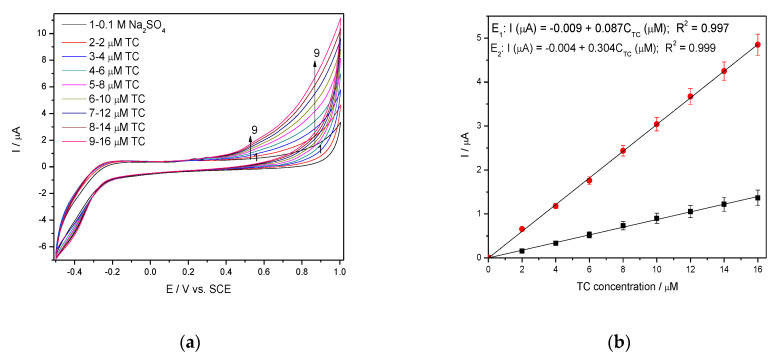
(**a**) CVs recorded at 0.050 V·s^−1^ scan rate on BDD/GO/Ag in 0.1 M Na_2_SO_4_ supporting electrolyte and in the presence of various TC concentrations: 2–16 µM TC; potential range: −0.500 to +1.00 V/SCE; (**b**) Calibration plots of the currents recorded at E_1_ = +0.550 V/SCE and E_2_ = +0.850 V/SCE vs. TC concentrations.

For comparison, the effect of the same 0.282 mM Cl^−^ concentration was tested for 0.1 M Na_2_SO_4_ sulfate for its considerationas a supporting electrolyte and the results presented in Figure 6b showed no effect, which means that Cl^−^ presence should not interfere the TC detection. This aspect was proved by comparative recording of the CV voltammograms series in 0.1 M Na_2_SO_4_ and in the presence of 0.282 mM Cl^−^ and the results are similar. Figure 7a,b present the results of TC detection at Ag/GO/BDD electrode in 0.1 M Na_2_SO_4_ supporting electrolyte. In comparison with 0.1 M NaOH, it is obvious that the results are different due to no silver oxides being formed.

The cyclic voltammograms recorded for the Ag/GO/BDD electrode in 0.1M Na_2_SO_4_ supporting electrolyte in the presence of increased TC concentrations are shown in Figure 7a and the calibration plots of anodic current and the TC concentrations are presented in Figure 7b.

In according with [27], TC oxidation occurred in two steps based on two electrons involvedat each step (Equations (5) and (6)).

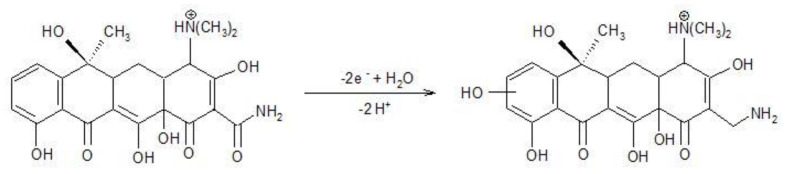
(5)

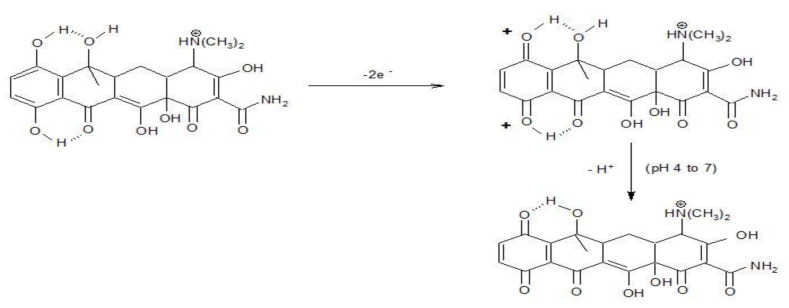
(6)

These steps allowed TC detecting at two potential values of +0.550 V/SCE and +0.850 V/SCE based on only anodic oxidation process. These two detection potential values are more positive than those anodic values found in 0.1 M NaOH supporting electrolyte (+0.266 V/SCE and +0.550 V/SCE), which shows the superiority of the alkaline medium usage. In addition, the presence of the cathodic detection potential in alkaline medium presents another advantage of the alkaline medium as supporting electrolyte. However, the best sensitivity was achieved at higher potential value of +0.850 V/SCE in sulfate electrolyte, which represents the advantage of sulfate electrolyte considering the lack of chloride interference as well. Within this context, further detection experiments will be carried out in both 0.1 M NaOH and 0.1 Na_2_SO_4_ supporting electrolytes.

#### 3.3.3. Differential-Pulsed Voltammetry (DPV) and Square-Wave Voltammetry (SWV) for TC Detection

Taking into account the main advantages of DPV and SWV techniques to mitigate the capacitive component of the background current, their utility for the enhancement TC detection was tested at different operating conditions in relation to the best sensitivity and lowest limit of detection in both 0.1 M NaOH and 0.1 Na_2_SO_4_ supporting electrolytes.

**Figure 8:**
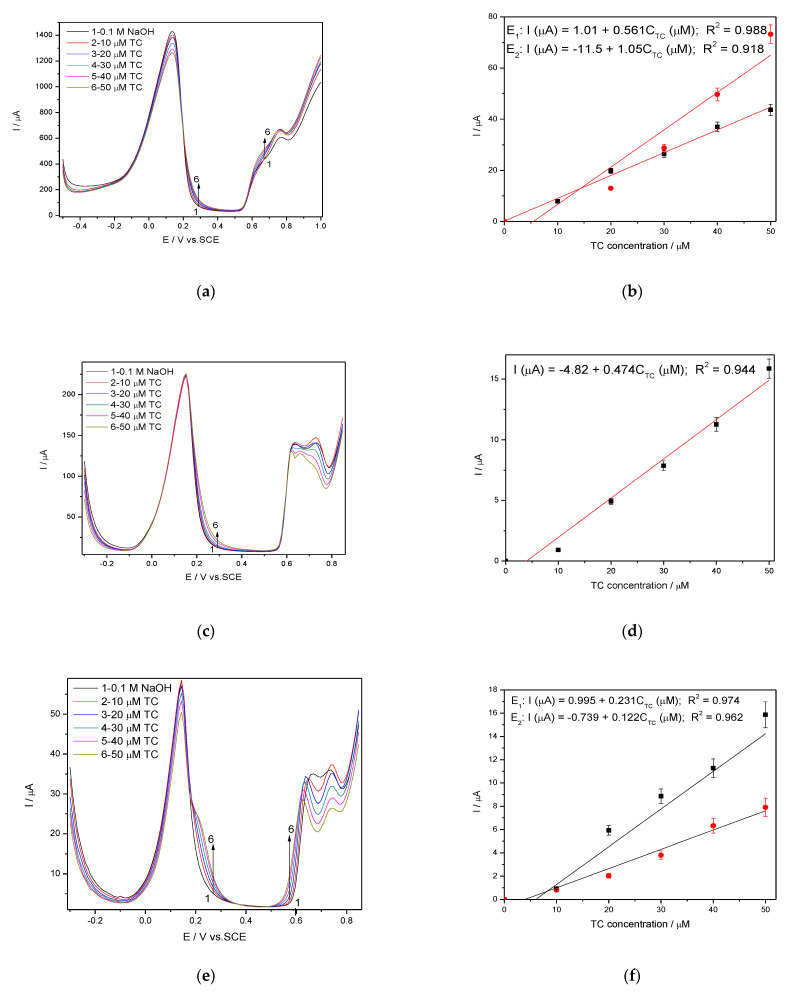
(**a**,**c**,**e**) Differential-pulsed voltammetry (DPV) recorded at the Ag/GO/BDD in 0.1 M NaOH supporting electrolyte and in the presence of various TC concentrations: 10–50 µM TC; step potential (SP) of 10 mV; modulation amplitude (MA) of 100 mV; potential ranges: −0.500 to +1.00 V/SCE (**a**); −0.350 to +0.850 V/SCE (**c**); −0.350 to +0.850 V/SCE with preconditioning at +1.00 V for 60s (**e**). Calibrations plots of the current vs. TC concentrations for corresponding operating conditions (**b**,**d**,**f**).

The selection of the modulation amplitude (MA), the step potential (SP) and the scan rate for the DPV technique considered the electrode stability and reproducibility among the electroanalytical performance. The response stability and the reproducibility was achieved for MA of 100 mV and the step potential of 10 mV at the scan rate of 0.050 V·s^−1^. A very interesting and different behaviour of the electrode was found for different potential range due to the silver redox system depending significantly on the potential scanning. The shapes of DPV recorded within the the potential range from −0.500 to +1.00 V/SCE that is similarly with CV testing and from −0.350 V to +0.850 V without/with preconditioning at +1.00 V/SCE for 60 s are shown in Figure 8a,c,e with the calibration plots presented in Figure 8b,d,f. The anodic peaks corresponding to silver oxidation to AgO are defined more or less in relation with the potential range, while the anodic peak corresponding to Ag_2_O formation is welldefined for all tested conditions, being sharpest for the potential range from −0.350 to +0.850 V after preconditioning to +1.00 V/SCE for 60 s. This last condition assured the most well-defined peaks for silver redox and shifted the detection potentials to be less positive than is desired in order to avoid the potential interferences but the sensitivities are lower in comparison with the potential range between −0.500 to +1.00 V/SCE (Table 3). This should be explained by the oxidation of TC through maintaining the potential value at +1.00 V/SCE for 60 min that leads to local TC concentration decreasing. However, the best lowest limit of detection was achieved under these operating conditions (0.120 µM) probably because of the better resolution even if the best sensitivity of 1.05 µA·µM^−1^ was achieved with the potential range from −0.500 to +1.00 V/SCE, which should be related to the larger current range.

**Figure 9:**
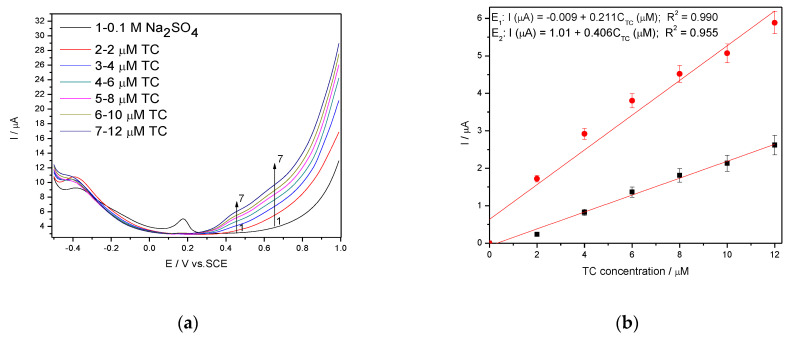
(**a**) DPVs recorded at the Ag/GO/BDD in 0.1 M Na_2_SO_4_ supporting electrolyte and in the presence of various TC concentrations: 2–10 µM TC; step potential (SP) 10 mV; modulation amplitude (MA) 100 mV; potential range: −0.500 to +1.00 V/SCE; (**b**) Calibration plots of the currents recorded at E_1_ = +0.433 V/SCE and E_2_ = +0.65 V/SCE versus TC concentrations.

The series of the DPVs recorded in 0.1 M Na_2_SO_4_ supporting electrolyte and TC concentrations ranged from 2 to 16 µM TC under the same operating conditions are presented in Figure 9a, and corresponding calibration plots presented in Figure 9b.

It can be noted that better electroanalytical performance in comparison with CV results is achieved, as we expected, but it is worse in comparison with DPV results achieved in 0.1 M NaOH supporting electrolyte.

**Figure 10:**
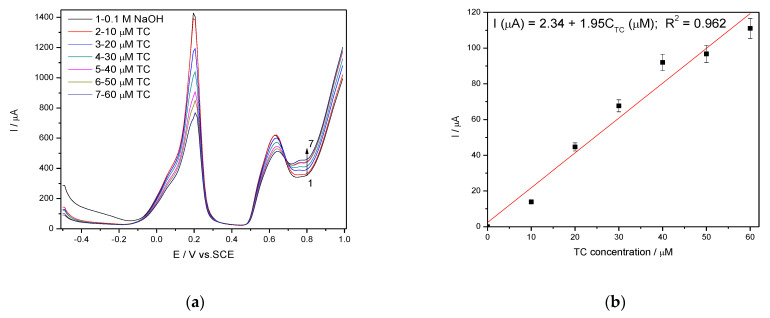
(**a**) Square-wave voltammetry (SWV) recorded on a Ag/GO/BDD in 0.1 M NaOH supporting electrolyte under optimized conditions at a frequency of 10 Hz, step potential (SP) 10 mV and modulation amplitude (MA) 200 mV in the potential range of −0.500 to +1.00 V vs. SCE in the presence of 10–60 µM TC concentration; (**b**) The calibration plots of the currents recorded at recorded at E = +0.766 V/SCE versus TC concentrations.

**Figure 11:**
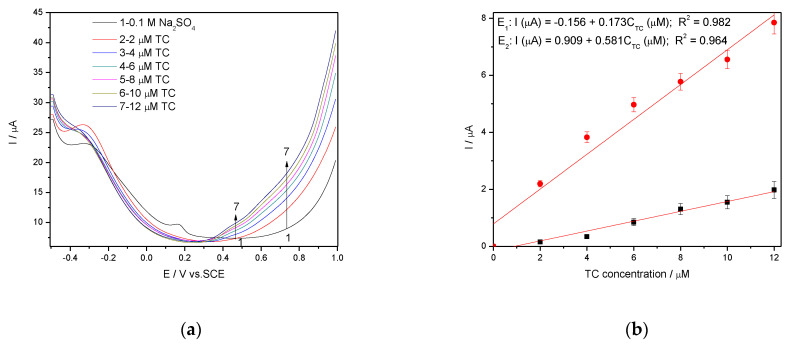
(**a**) SWV recorded on a Ag/GO/BDD in 0.1 M Na_2_SO_4_ supporting electrolyte under optimised conditions at a frequency of 10 Hz, step potential (SP) 10 mV and modulation amplitude (MA) 100 mV in the potential range of −0.500 to +1.00 V vs. SCE in the presence of a 2–12 µM TC concentration; (**b**) The calibration plots of the currents recorded at recorded at E_1_ = +0.436 V/SCE and E_2_ = +0.670 V/SCE versus TC concentrations.

It is wellknown that SWV is a large-amplitude differential technique that exhibit the advantage of its speed [28,29,30,31], controlled by the product of frequency (f) and step potential (SP). The potential amplitude of 100 mV tested in DPV was not suitable for SWV application at Ag/GO/BDD in 0.1 M NaOH supporting electrolyte related to the detection application because no response in the presence of TC was achieved. The larger amplitude of 200 mV with the step potential of 10 mV and 5 mV, and the frequency of 10 Hz allowed the response achievement, and the results reached at SP of 10 mV aregiven in Figure 10a,b. The best electroanalytical performance considering all parameters (detection potential value, the sensitivity and LOD) was achieved for the step potential of 5 mV. However, in 0.1 M Na_2_SO_4_ supporting electrolyte, Ag/GO/BDD electrode did not give a stable response for the modulation amplitude of 200 mV and the series of SWV recorded at 100 mV are shown in Figure 11a, which allowed achieving the linear dependence of the current vs. TC concentrations at the potential value of +0.436 V/SCE and, respectively, +0.670 V/SCE (Figure 11b).

Worse electroanalytical parameters in 0.1 M Na_2_SO_4_ in comparison with 0.1 M NaOH can be noted, but the more negative detection potential is favorable in terms of interfering aspects. We tested 0.1 M Na_2_SO_4_ supporting electrolyte-based procedure using SWV operated at modulation amplitude of 100 mV and the step potential of 10 mV using Ag/GO/BDD electrode for tap water spiking with the same TC concentration range and similar results regarding the electroanalytical performance were reached, which validated the results for the practical applications (Table 4).

#### 3.3.4. Chrono and Multiple-Pulsed Amperometry for Tetracycline Detection

**Figure 12:**
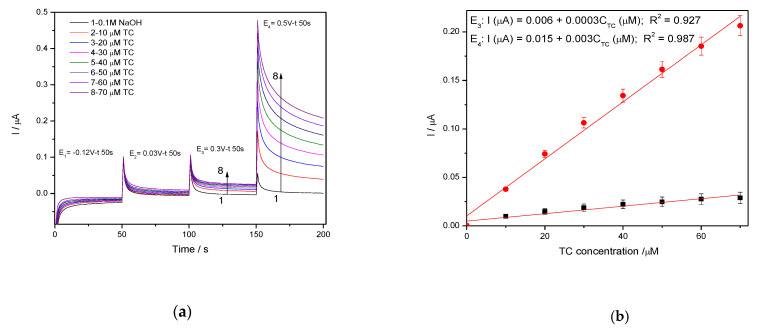
(**a**) Chronoamperometry(CA) recorded on Ag/GO/BDD electrode at the potential value of E_1_ = −0.150 V/SCE, E_2_ = +0.030 V/SCE, E_3_ = +0.300 V/SCE and E_4_ = +0.500 V/SCE in 0.1 M NaOH supporting electrolyte and in the presence of 10 to 70 µM TC. (**b**) The calibration plots of the currents recorded at E_3_ = +0.3 V/SCE and E_4_ = +0.500 V/SCE vs. TC concentrations.

**Figure 13:**
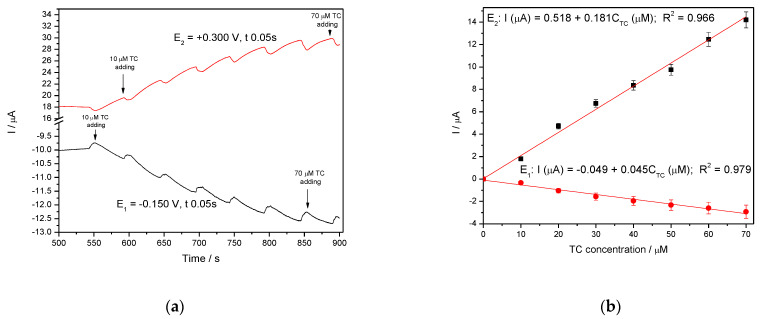
(**a**) Multiple-pulsed amperometry (MPA) recorded at Ag/GO/BDD electrode in 0.1 M NaOH supporting electrolyte, in the presence of 10–70 µM TC concentration: 10 µM, 20 µM, 30 µM, 40 µM, 50 µM, 60 µM, 70 µM at two applied potential levels, i.e., E_1_ = +0.300 V/SCE and E_2_ = −0.150 V/SCE. (**b**) Calibration plots for tetracycline detection in the concentration range 10–70 µM recorded at the potential E_1_ = −0.150 V/SCE and E_2_ = +0.300 V/SCE.

**Figure 14:**
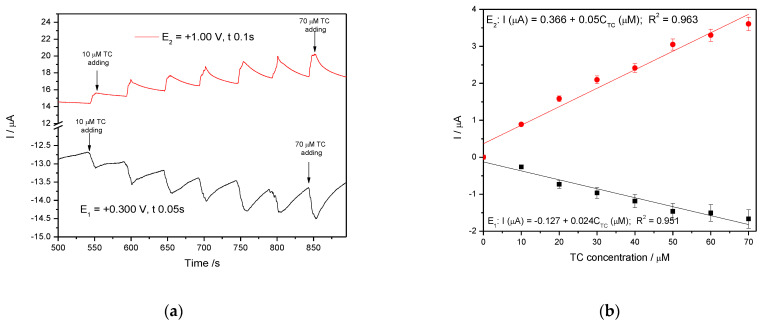
(**a**) MPAs recorded at Ag/GO/BDD electrode in 0.1 M NaOH supporting electrolyte, in the presence of 10–70 µM TC concentration: 10 µM, 20 µM, 30 µM, 40 µM, 50 µM, 60 µM, 70 µM at two applied potential levels, i.e., +0.300 V/SCE and +1.00 V/SCE. (**b**) Calibration plots for tetracycline detection in the concentration range 10–70 µM recorded at the potential E_1_ = +0.300 V/SCE and E_2_ = +1.00 V/SCE.

**Figure 15:**
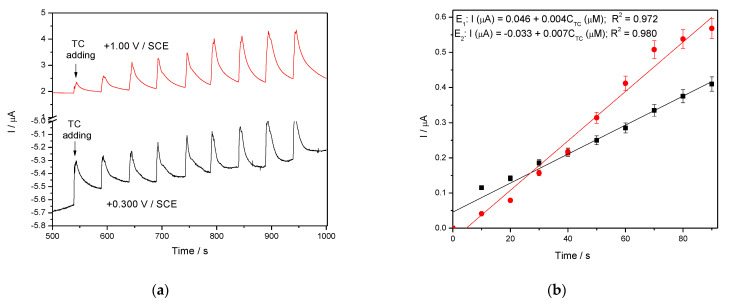
(**a**) MPAs recorded at Ag/GO/BDD in 0.1 M Na_2_SO_4_ supporting electrolyte in the presence of various TC concentrations: 10–90 µM TC at two applied potential levels, +0.300 V/SCE and +1.00 V/SCE. (**b**) The calibration plots of the currents recorded at recorded at E_1_ = +0.300 V/SCE and E_2_ = +1.00 V/SCE versus TC concentrations.

In general, amperometry represents the easiest electrochemical method for practical detection and one/multi-levels-based CA can be applied to enhance the electroanalytical parameters, considering CV behavior as reference. Four levels of chronoamperograms were recorded at the potential values of −0.150 V/SCE, +0.030 V/SCE, +0.300 V/SCE and +0.500 V/SCE corresponding to the silver oxides-redox system and all anodic currents increased at each potential level; the best sensitivity was achieved at the potential value of +0.500 V/SCE (Figure 12a,b), which is much lower in comparison with the voltammetric techniques. A pseudo MPA technique reduced to double-pulsed amperometry was proposed considering two potential levels at −0.150 V/SCE and +0.300 V/SCE corresponding to the silver (I) oxides redox system and the amperograms are presented in Figure 13b. This proposed technique assured very good electroanalytical performances which are comparable with those obtained by CV, which should explain by fast passing from the oxidation of silver (I) oxides to its reduction, which are involved in TC oxidation and its oxidation product reduction assuring in-situ activation of redox system. If the two detection potential levels are selected at +0.300 V/SCE and +1.00 V/SCE considering the silver (I) and silver (II) oxides (Figure 14a,b), the electroanalytical performances are better than those obtained for four-level chronoamperograms but worse in comparison with those achieved at the potential levels of −0.150 V/SCE and +0.300 V/SCE. The similar double-pulsed amperometry operated at +0.300 V/SCE and +1.00 V/SCE recorded in 0.1 M Na_2_SO_4_(Figure 15a,b) led to worse electroanalytical performances in comparison with 0.1 M NaOH.

The electroanalytical performances for TC detection obtained by all studied electrochemical voltammetric and amperometric techniques in both 0.1 M NaOH and 0.1 M Na_2_SO_4_ supporting electrolytes are gathered in Table 5. The SWV technique achieved the best sensitivity and LOD and LQ at a more positive potential value (+0.830 V vs. SCE). The good results reached for MPA appliedat two potential levels of −0.150 V/SCE and +0.300 V/SCE should be taken into account due to the method’s simplicity and lower negative detection potential value that mitigates the interference aspects.

The comparison of the lowest limit of detection obtained with this modified Ag/GO/BDD electrode by SWV with other previous works is presented in Table 6, and the superiority of the voltammetric detection procedure proposed in this work can be seen.

In addition, the recovery test was performed by analyzing three parallel surface water samples, which were spiked with 10, 50 and 100 µg·L^−1^ TC. The recoveries test was run in the same 0.1 M Na_2_SO_4_ supporting electrolyte using SWV operated at MA of 100 mV, the step potential of 10 mV, and frequency of 10 Hz. The recovery values higher than 95% and the RSD values smaller than 5% for all three concentrations indicated good recovery and reproducibility of the results and the great potential of the Ag/GO/BDD for water quality monitoring related to the quantitative determination of the tetracycline and other types of application such as quantitative determination in pharmaceutical formulation. Repeatability of the proposed detection procedure was evaluated by comparing the results of the determination of a solution containing 50 µg·L^−1^ TC during three days. The relative standard deviation less than 5% demonstrated a good repeatability of the proposed Ag/GO/BDD electrode-based voltammetric procedure.

## 4. Conclusions

In this study the importance of the electrode carbon substrate as host for the silver electrodeposition within a BDD-based modified electrode characterized by the electrocatalytic effect towards TC oxidation and the oxidation product reduction, which determined its detection, was proved. In addition, the great role of the supporting electrolyte as alkaline or slight acidic pH was demonstrated in direct relation with the electrochemical behaviour of silver on the carbon electrode substrate. CV results allowed to select BDD electrode modified with graphene oxide reduced electrochemically and further decorated with silver named Ag/GO/BDD electrode as the most suitable among a series of BDD, graphene oxide reduced onto BDD, and silver electrodeposited onto BDD electrodes. All electrochemical voltammetric and amperometric techniques tested were appropriate for the development of the electrochemical detection procedure for the quantitative determine of TC in alkaline aqueous solution. The best results regarding the sensitivity (46.6 µA·µM^−1^·cm^−2^) and the lowest limit of detection (5 nM) was achieved using SWV operated at the step potential of 5 mV, modulation amplitude of 200 mV and the frequency of 10 Hz in alkaline medium. Also, a pseudo-MPA technique reduced to simple double-pulsed amperometry operated at two potential levels of −0.150 V/SCE and +0.300 V/SCE allowed very nice results for TC detection. However, the application of this alkaline medium based procedure for water monitoring is limited by the presence of chloride that interfere with TC detection, but it can be applied for quantitative determination of pharmaceutical formulations. For water monitoring application, Ag/GO/BDD can be used for TC detection in 0.1 M Na_2_SO_4_ supporting electrolyte with the SWV technique operated under SP of 10 mV, MA of 100 mV and frequency of 10 Hz.

Based on the results of this study including the reproducibility and the repeatability of the proposed method, it can be concluded that BDD electrode modified with graphene oxide reduced electrochemically and further decorated with silver is appropriate for the electrochemical detection of tetracycline in water using a 0.1 M Na_2_SO_4_ supporting electrolyte and in pharmaceutical formulations using a 0.1 M NaOH supporting electrolyte.

## Figures and Tables

**Table 1:** Comparative apparent diffusion coefficient and electroactive surface area.

Electrode	Apparent Diffusion Coefficient ·10^6^/cm^2^·s^−1^	Electroactive Surface Area/cm^2^
BDD	1.54	0.037
GO/BDD	1.60	0.038
Ag/BDD	1.91	0.041
Ag/GO/BDD	2.50	0.045

**Table 2:** Comparative detection potential values and the sensitivities.

Electrode Type	Potential Range	E_det_ (V/SCE)	Sensitivity (µA·µM^−1^·cm^−2^)
BDD	Anodic	+0.700	0.085
GO/BDD	Anodic	+0.700	0.076
Ag/BDD	Anodic	+0.285	3.0
		+0.550	1.03
Ag/GO/BDD	Anodic	+0.296	3.17
		+0.550	2.86
	Cathodic	+0.039	2.16

**Table 3:** The electroanalytical parameters obtained using differential-pulsed voltammetry (DPV) at a MA of 100 mV and the SP of 10 mV.

Operating Conditions		Electroanalytical Performance		
Potential Range/V	Preconditioning	E_det_ (V/SCE)	Sensitivity (µA/µM·cm^−2^)	Lowest Limit of Detection, LOD (µM)
−0.500 → +1.00	-	+0.300	8.01	0.224
		+0.640	15.0	0.139
−0.350 → +0.850	-	+0.260	6.77	0.221
−0.350 → +0.850	+1.00 V for 60s	+0.220	3.30	1.41
		+0.566	1.74	0.120

**Table 4:** The analytical parameters obtained using square wave voltammetry at frequency of 10 Hz.

Operating Conditions		Electroanalytical Performance		
**Supporting Electrolyte**	MA (mV)/ SP (mV)	E_det_ (V/SCE)	Sensitivity (µA/µM·cm^−2^)	Lowest Limit of Detection, LOD (µM)
0.1 M NaOH	200/10	+0.766	27.9	0.101
	200/5	+0.750	46.6	0.035
0.1 M Na_2_SO_4_ *	100/10	+0.436	2.47	0.623
		+0.670	8.30	0.155

* tap water spiked with TC concentration.

**Table 5:** Electroanalytical performance of Ag/GO/BDD electrode for TC detection in aqueous solution.

Supporting Electrolyte	Technique	Conditions	E_det_ (V/SCE)	Sensitivity (µA·µM·cm^−2^)	R^2^	LOD ^1^ (µM)	LQ ^2^ (µM)	RSD ^3^ (%)
0.1 M NaOH	CV	v = 0.05 V·s^−1^	+0.296	3.17	0.990	1.22	4.08	3.15
			+0.550	2.86	0.966	1.10	3.68	4.71
			−0.039	2.16	0.908	0.100	0.330	1.84
	DPV	SP 10 mV,	+0.300	8.01	0.988	0.224	0.748	2.58
		MA 100 mV	+0.640	15.0	0.918	0.013	0.464	0.768
	SWV	SP 5 mV, MA 200 mV, f 10 Hz	+0.750	46.6	0.969	0.005	0.017	0.135
	CA	4 levels	+0.300	0.004	0.927	0.567	1.89	6.25
			+0.500	0.043	0.987	0.500	1.770	9.25
	MPA	t 0.05 s	−0.150	0.643	0.979	0.670	2.22	0.100
		t 0.05 s	+0.300	2.59	0.972	0.160	0.56	0.126
		t 0.05 s	+0.300	0.343	0.951	1.47	4.91	0.145
		t 0.05 s	+1.00	0.714	0.963	1.36	4.54	0.158
0.1 M Na_2_SO_4_	CV	v = 0.05 V·s^−1^	+0.550	1.24	0.997	1.05	3.51	4.59
			+0.830	4.34	0.999	0.536	1.79	3.79
	DPV	SP 10 mV,	+0.433	3.01	0.990	0.350	1.17	0.797
		MA 100 mV	+0.650	5.80	0.955	0.216	0.722	0.763
	SWV	SP 10 mV, MA 100 mV, f 10 Hz	+0.436	2.47	0.982	0.423	2.08	0.483
			+0.670	8.30	0.964	0.155	0.516	0.368
	SWV water tap	SP 10 mV, MA 100 mV, f 10 Hz	+0.436	2.50	0.980	0.194	0.640	0.297
			+0.670	8.24	0.975	0.230	0.768	0.709
	MPA	t 0.05 s t 0.05 s	+0.3V	0.060	0.972	1.89	6.29	0.260
			+1V	0.100	0.980	2.45	8.16	0.716

^1,2^ The lowest limit of detection and the lowest limit of quantification, respectively, determined in accordance with the literature [26]; ^3^ For three replicates.

**Table 6:** Comparison of the lowest limit of detection (LOD) obtained in this study with other reported works.

Modified Electrode	LOD (µM)	Reference
Screen-printed carbon electrodes modified with imprinted overoxidized polypyrrole (MIOPPY) and gold nanoparticles (AuNP)	0.650	[3]
Carbon paste electrode modified with a combination of multiwalled carbon nanotubes functionalized with carboxyl groups	0.360	[11]
Benzene sourced graphene-gold nanoparticle sensor	0.160	[12]
Multisegment nanoparticles	15.2	[13]
Reduced graphene oxide and magnetite nanoparticles	0.585	[23]
Boron-doped diamond electrode modified with CoAl_2_O_4_	0.500	[24]
Electrodeposited carbon nanotubes on gold coated glassy carbon electrode	0.0945	[25]
Graphite-polyurethane composite electrode	2.80	[32]
Multi-wall carbon nanotube modified glassy carbon electrode (MWCNT-GCE)	0.810	[33]
Silver decorated graphene oxide reduced onto boron-doped diamond (Ag/GO/BDD)	0.005	This work

## Data Availability

Not applicable.
